# Challenges and Controversies in the Surgical Treatment of Cervical Cancer: Open Radical Hysterectomy versus Minimally Invasive Radical Hysterectomy

**DOI:** 10.3390/jcm10173761

**Published:** 2021-08-24

**Authors:** Jona Röseler, Robert Wolff, Dirk O. Bauerschlag, Nicolai Maass, Peter Hillemanns, Helder Ferreira, Marie Debrouwere, Fülöp Scheibler, Friedemann Geiger, Mohamed Elessawy

**Affiliations:** 1 Department of Gynecology and Obstetrics, University Hospital Schleswig-Holstein, Campus Kiel, 24105 Kiel, Germany; Dirk.Bauerschlag@uksh.de (D.O.B.); Nicolai.Maass@uksh.de (N.M.); Mohamed.elessawy@uksh.de (M.E.); 2 Kleijnen Systematic Reviews Ltd., Escrick, York YO19 6FD, UK; Robert@systematic-reviews.com; 3 Department of Gynecology and Obstetrics, Hannover Medical School, 30625 Hannover, Germany; Hillemanns.Peter@MH-Hannover.de; 4 Department of Gynecology, Centro Hospitalar, University of Porto, 4099-001 Porto, Portugal; helderreira@hotmail.com; 5 National Competency Center for Shared Decision Making, University Hospital Schleswig-Holstein, 24105 Kiel, Germany; Marie.Debrouwere@iqwig.de (M.D.); Fueloep.Scheibler@uksh.de (F.S.); F.Geiger@uksh.de (F.G.)

**Keywords:** cervical cancer, early-stage cervical cancer, LACC trial, minimally invasive surgery, open radical hysterectomy

## Abstract

Objective: The aim of the study was to perform a systematic assessment of disease-free survival (DFS), overall survival, and morbidity rates after open radical hysterectomy (ORH) and minimally invasive surgery (MIS) for early-stage cervical cancer and discuss with experts the consequences of the LACC trial (published by Ramirez et al. in 2018) on clinical routine. Methods: A total of 5428 records were retrieved. After exclusion based on text screening, four records were identified for inclusion. Five experts from three independent large-volume medical centers in Europe were interviewed for their interpretation of the LACC trial. Results: The LACC trial showed a significantly higher risk of disease progression with MIS compared to ORH (HR 3.74, 95% CI 1.63 to 8.58). This was not seen in one epidemiological study and was contradicted by one prospective cohort study reported by Greggi et al. A systematic review by Zhang et al. mentioned a similar DFS for robot-assisted radical hysterectomy (RRH) and LRH. Recurrence rates were significantly higher with MIS compared to ORH in the LACC trial (HR 4.26, 95% CI 1.44 to 12.60). In contrast, four studies presented by Greggi reported no significant difference in recurrence rates between LRH/RRH and ORH, which concurred with the systematic reviews of Zhang and Zhao. The experts mentioned various limitations of the LACC trial and stated that clinicians were obliged to provide patients with detailed information and ensure a shared decision-making process. Conclusions: The surgical treatment of early-stage cervical cancer remains a debated issue. More randomized controlled trials (RCT) will be needed to establish the most suitable treatment for this condition.

## 1. Introduction

In 2018, Ramirez et al. published the results of a randomized controlled trial (RCT) on the laparoscopic approach to cervical cancer (LACC) in the New England Journal of Medicine [1]. This non-inferiority trial evaluated survival rates after minimally invasive surgery (MIS) versus open radical hysterectomy (ORH) for early-stage cervical cancer (FIGO stage 2009 IA_1_ with lymphovascular invasion, IA_2_, or IB_1_). The authors concluded that minimally invasive radical hysterectomy was associated with lower rates of overall survival and disease-free survival compared to ORH [1].

These data had a striking impact on gynecological surgery throughout the world. Since the minimally invasive approach had become the more favorable technique due to its less severe side effects and equivalent oncological outcome compared to other procedures, the results of the LACC trial were unexpected [2,3,4,5]. Despite some issues regarding the implementation of the LACC trial, the ESMO issued an amendment of their guidelines, by which radical hysterectomy performed by laparoscopy or robot-assisted surgery may no longer be given preference over open surgery for patients with FIGO stage 2019 IA_2_, IB, and IIA [6,7].

Given the numerous issues addressed in the LACC trial, the inferiority of the minimally invasive approach in all cases remains controversial [8,9,10,11]. However, once the results of the LACC trial were published, it became difficult to justify the implementation of any new RCT on the same issue.

We collected data from a wide range of studies evaluating the difference between ORH and minimally invasive radical hysterectomy, performed a review of published reviews, summarized the results, and compared these with the outcome of the LACC trial. Furthermore, we collected expert opinions from renowned clinicians.

## 2. Methods

The objective was to compare the results of the LACC trial with those reported in the published literature on the efficacy of MIS versus ORH in early-stage cervical cancer. Based on an extensive literature research, we screened 3966 publications that dealt with the surgical treatment of early-stage cervical cancer. We extracted data from three concise reviews published from 2017 to 2020, including 63 primary studies.

This literature review was carried out according to the methods recommended by Cochrane [12] and the Center for Reviews and Dissemination (CRD) [13], in alignment with the PRISMA reporting guidelines [14,15]. No ethical approval was required.

### 2.1. Study Selection

Key inclusion criteria were defined using a PICOS (population, intervention, comparator, outcome, study design) approach. After an initial needs assessment of patients based on their priorities and experience, we defined prespecified outcomes of interest.

- Population: patients with early-stage cervical cancer
- Intervention/comparator: ORH, MIS, robotic radical hysterectomy (RRH), and/or laparoscopic radical hysterectomy (LRH)
- Outcomes: overall survival (OS), disease-free survival (DFS), measures of surgery (e.g., operating time, blood loss, length of hospital stay), rates of intra and postoperative complications
- Study design: systematic reviews (SRs), meta-analyses, literature reviews, and randomized controlled trials (RCTs)

Titles and abstracts identified through the electronic database and web searches and, subsequently, full paper copies of all potentially eligible reports were screened independently by two reviewers; any disagreements were resolved by discussion. No restrictions were placed on language or publication status.

In order to compare the existing (non-randomized) evidence with the LACC trial conducted by Ramirez et al., newer reviews covering comparable outcomes were selected.

### 2.2. Searches

To avoid missing any relevant SRs and RCTs, a focused literature search was conducted using a combination of free text and database thesaurus terms. No restrictions on language or publication status were applied. Reference lists of the included articles were also searched for additional studies.

Relevant RCTs were searched in the following databases from 2014 to March 2020: MEDLINE (Ovid): 1946-2020/03/02, MEDLINE In-Process Citations, Medline Daily Update and Epub Ahead of Print (Ovid): to 2 March 2020, Embase (Ovid): 1974-2020/03/02, Cochrane Central Register of Controlled Trials (CENTRAL) (Wiley): to 2020/03/Iss3. Relevant SRs were searched in the following resources from 2014 to March–May 2020: Cochrane Database of Systematic Reviews (CDSR) (Wiley): to 2020/03/Iss3, KSR Evidence (KSR): to 2020/03/03, PROSPERO (CRD): to 5 May 2020, MEDLINE (Ovid): 1946-2020/05/08, MEDLINE In-Process Citations, Medline Daily Update and Epub Ahead of Print (Ovid): to 8 May 2020, Embase (Ovid): 1974-2020/05/08. An example of the search strategy is reported in Appendix A.

### 2.3. Data Extraction

Data were extracted by one reviewer and checked by a second. Any discrepancies were resolved by discussion or the intervention of a third reviewer.

### 2.4. Reviewing Expert Opinions from Large-Volume Centers

Oncologists from three independent medical centers in Europe servicing large numbers of patients and adhering to a certified mode of quality control were interviewed. The questionnaire addressed their experience after the results of the LACC trial. All experts were confronted with the data of the literature research performed in this study. These findings have been included in the discussion. Detailed answers can be found in Appendix A.

### 2.5. Quality (Risk of Bias) Assessment

The risk of bias (methodological quality) of RCTs was assessed using the Cochrane Risk of Bias Tool for randomized clinical trials, while reviews were assessed using ROBIS [16,17].

Risk of bias assessment was performed by one reviewer and checked by a second reviewer. Any discrepancies were resolved by the intervention of a third.

### 2.6. Data Synthesis

Results of the LACC trial and relevant reviews were reported as a narrative synthesis with tables and figures.

## 3. Results

### 3.1. Literature Searches and Inclusion Assessment

A total of 5428 records were retrieved from the database searches. After de-duplication, 3996 records remained. These articles were screened on the basis of titles and abstracts; 13 were ordered for full-text screening and eight were excluded. After the full-text screening stage, three records were identified for inclusion in the review.

**Figure 1:**
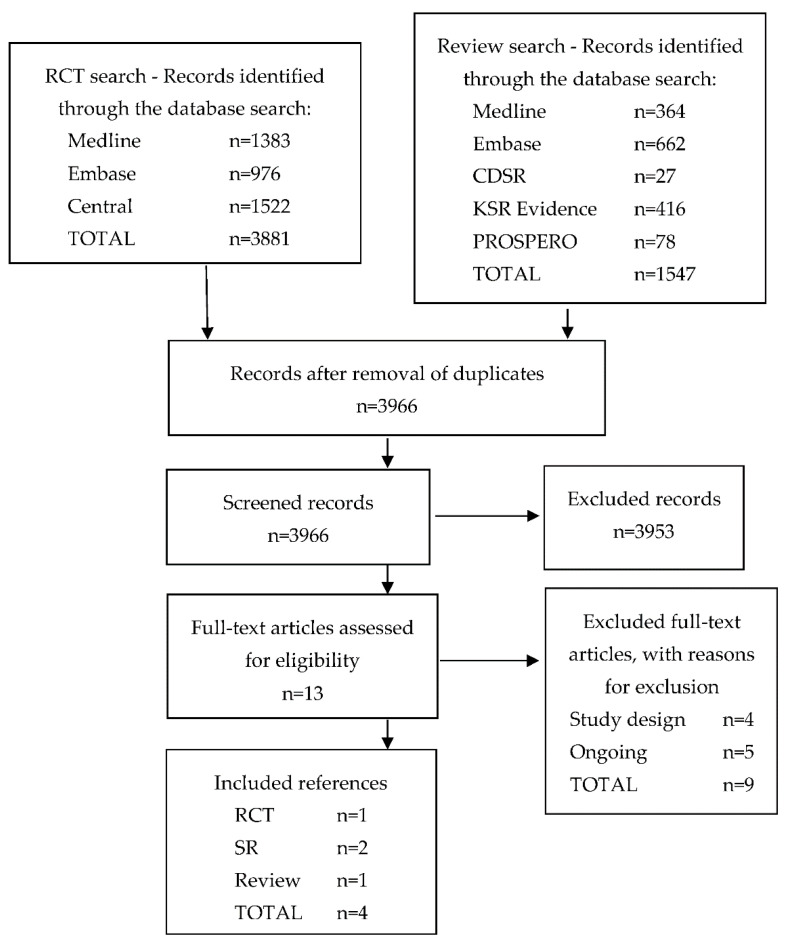
Flow chart—selection process. CDSR = Cochrane Database of Systematic Reviews; KSR = Kleijnen Systematic Reviews; NMA = network meta-analysis; PROSPERO = International Prospective Register of Systematic Reviews; RCT = randomized controlled trial; SR = systematic review.

The flow of studies through the search and screening processes is summarized in Figure 1.

### 3.2. Overview of Included References

This review included four recent publications (Table 1), including one RCT, the Laparoscopic Approach to Cervical Cancer (LACC) trial, published in November 2018 [1,18,19]. Greggi et al. [20] was a recent narrative review, while Zhao and Zhang were both SRs [21,22].

The primary studies included in the reviews are listed in Table A1 (see Appendix A). A similar number of primary studies were included in each review, i.e., Zhao 2017 (*n* = 23), Zhang 2019 (*n* = 25), and Greggi (*n* = 24).

A total of 63 primary studies were incorporated in the three reviews. None of these studies were included in all three secondary publications, while nine primary studies featured in two of the secondary publications. The reasons for these were the eligibility criteria and the methodology of the reviews.

### 3.3. Methods of LACC Trial

The LACC trial was an international, open-label, multicenter phase III RCT evaluating the hypothesis that minimally invasive (laparoscopic or robot-assisted) radical hysterectomy was not inferior to ORH. The trial was conducted at 33 centers across the world and recruited patients between June 2008 and June 2017.

The primary outcome was DFS or death from cervical cancer. Secondary outcomes were locoregional recurrence (pelvic or distal), progression-free survival, treatment-related morbidity (intraoperative complications, time to discharge, perioperative complications, early postoperative wound and vault complications within 4 weeks, long-term morbidity to 6 months, estimated blood loss, postoperative pain, and analgesic consumption), OS, quality of life, pelvic floor distress inventory, feasibility of sentinel lymph node biopsy, and costs.

### 3.4. Results of the LACC Trial

The trial was terminated prematurely in June 2017 by the data and safety monitoring committee due to concerns about an imbalance in deaths between the two groups. A total of 631 patients had been randomized until this time: 319 to minimally invasive and 312 to ORH. In the minimally invasive group, 269 (84.4%) underwent laparoscopy and 50 (15.6%) underwent robot-assisted surgery.

The estimated rates of DFS at 4.5 years were 86.0% for MIS and 96.5% for ORH (difference −10.6%, 95% confidence interval (CI) −16.4 to −4.7%). There was no evidence of the non-inferiority of minimally invasive surgery to ORH, as the 95% CI included the non-inferiority margin of −7.2%. DFS following MIS was significantly poorer than with ORH (Table 2).

Adverse events of the LACC trial were reported by Obermair et al., including the incidence of intra and postoperative adverse events within six months after surgery [23]. This analysis included 536 (85%) of the 631 randomized patients, of whom 279 (52%) underwent MIS and 247 (48%) ORH. Only those who had undergone surgery and been followed up for at least six months were included in the analysis, which was based on the treatment received (Table 3).

### 3.5. Overview of the Results

Table 4 compares outcomes, DFS (disease-free survival), overall survival (OS), and recurrence rates, in the LACC trial and the three secondary publications.

The LACC trial revealed a significantly higher risk of disease progression or death with MIS compared to ORH (HR 3.74, 95% CI 1.63 to 8.58). However, the increase was not seen in two meta-analyses and one epidemiological study reported in the review by Greggi et al., which yielded no significant difference between laparoscopic radical hysterectomy (LRH) and ORH. One prospective cohort study contradicted the LACC and reported a DFS of up to 5 years with MIS compared to ORH (92.8 vs. 81.3%, *p* = 0.03) [20]. The SR performed by Zhang et al. included seven observational studies reporting either the estimated percentage with DFS or the disease-free interval. Although no meta-analysis was performed, the authors registered similar DFS rates for RRH and LRH, and a slightly higher DFS rate for RRH compared to ORH [22].

The numbers of patients with recurrent disease were also significantly higher after MIS compared to ORH in the LACC trial (HR 4.26, 95% CI 1.44 to 12.60). In contrast, the four studies reviewed by Greggi et al. that reported recurrence rates yielded no significant difference between RRH or LRH and ORH [20]. The SRs performed by Zhang and Zhao also did not reveal any significant difference in recurrence rates between the various types of surgery [21,22].

As regards overall survival, the LACC trial showed a significantly higher risk of death due to any cause after MIS compared to ORH (93.8 vs. 99.0%; HR 6.00, 95% CI 2.77 to 20.3) [1]. Greggi et al. mentioned one prospective study that reported significantly better survival rates after MIS compared to ORH (91 vs. 78.9%, *p* = 0.026), and one epidemiological study that reported a significantly lower survival rate after LRH compared to ORH (90.9 vs. 94.7%, *p* = 0.002) [20]. The two other secondary publications reporting on overall survival did not mention a statistically significant difference between LRH and ORH (Table 4).

Zhao noted significantly fewer pelvic lymph nodes retrieved with LRH compared with ORH (mean difference (MD) 1.65, 95% CI 3.19 to 0.11), but Zhang found no significant difference between RRH and ORH.

The estimated blood loss was significantly lower for MIS compared to ORH in the LACC trial [1]. Similar data were noted by Zhao and Zhang in their SRs [21,22]. Accordingly, fewer patients with MIS required a blood transfusion compared to those who underwent ORH in the LACC trial as well as in the SRs by Zhao and Zhang [1,21,22]. The systematic review by Zhao reported significantly fewer patients with adverse events such as ileus or vaginal fistulas after LRH compared to ORH, while the LACC trial reported no significant difference between groups (Table 5).

### 3.6. Risk of Bias

Overall, the risk of bias was rated low for the LACC trial [16]. Some minor issues were noted, such as blinding and reporting of the safety population.

The publication by Greggi et al. was a narrative review and hence did not follow a predefined protocol or inclusion criteria. This publication has been assigned an unclear overall risk of bias for each domain [20]. The risk of bias was rated high for the SRs performed by Zhang and Zhao.

**Figure 2:**
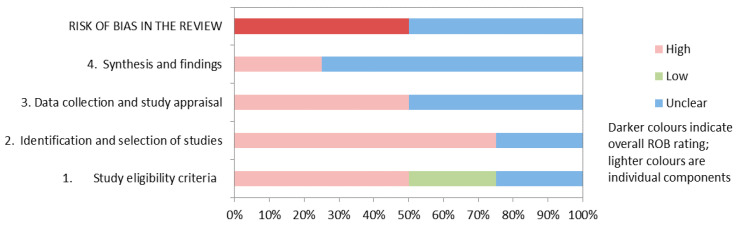
Risk of bias assessment of the identified reviews. Based on Whiting et al., 2016 [17], ROB = risk of bias. The table shows the risk of bias in the included references.

The main limitation of both SRs was that they were based on observational studies, with the exception of one small Chinese RCT included in Zhao’s review [21,22,25,26]. Many of the meta-analyses reviewed by Zhang and Zhao had high levels of statistical heterogeneity, which may have been due to the fact that they pooled a variety of prospective and retrospective study designs [21,22]. Neither of the reviews included a sensitivity or subgroup analysis, particularly by study design, to explore this heterogeneity. A summary is shown in Figure 2, while detailed assessments of the three secondary publications are reported in Supplementary Table S1.

## 4. Discussion

The data of the three reviews highlight the discrepancy between the LACC trial and the expectations of gynecologists throughout the world, namely the fact that MIS may worsen the outcome.

This discrepancy characterizes the still ongoing dilemma faced by experts. The minimally invasive approach had become the more favored technique because of its fewer side effects and estimated equivalent oncological outcome compared to other procedures for the treatment of early-stage cervical cancer [8,9,10].

The outcomes presented in the LACC trial, i.e., DFS, OS, recurrence rates, and adverse events, were extracted for all reviews. Zhang et al. reported RRH as a unique intervention, while Zhao and Greggi et al. summarized RRH and LRH as MIS [20,21,22]. In contrast to the main endpoint of the LACC trial, the three reviews did not report a significantly lower DFS after MIS compared to ORH [20,21,22]. Observational studies mentioned by Zhang et al. even described a higher DFS with RRH compared to ORH [20,21,22].

The same was noted for the secondary endpoints of OS and recurrence rates; neither of these differed significantly between MIS and ORH in all three reviews [20,21,22].

In the LACC trial, MIS was associated with shorter hospital stays, less blood loss, and less blood transfusion but higher intra and postoperative complication rates. The reviews of Zhao and Zhang et al. provided a more detailed account of these aspects. Zhao reported significantly fewer pelvic lymph nodes retrieved with LRH compared with ORH, but Zhang found no significant difference between RRH and ORH.

These findings corroborate the personal experience of the experts we addressed from University Hospital Center of Porto, Portugal, Hannover Medical School, and University Hospital Kiel, Germany, who failed to confirm a significant difference in DFS and the risk of death between ORH and LRH at their medical centers.

The main objection to the LACC trial was its short duration of follow-up, which may have concealed a higher recurrence rate in the ORH group [10]. Furthermore, the proficiency of surgeons in performing MIS is debatable because the surgeons needed to have conducted only ten operations in order to participate in the trial. Additionally, the participation rate of countries in which MIS is a highly established method was low [9,10]. The expert at the Hannover medical school stated that “some critics question the operative expertise of the respective centers, especially with reference to the learning curve of laparoscopic radical hysterectomy, which became established as a routine procedure worldwide only a few years before the start of the study”.

One of the main points of criticism about the LACC trial is the heterogeneous use and missing evaluation of the uterine manipulator. This point was addressed in earlier studies. Breakage of the uterine manipulator in the pelvis or shortly after surgery is responsible for many cases of recurrence, which was probably also true of the LACC trial [10,26,27]. Chiva et al. performed a large retrospective multicenter trial in Europe and evaluated the role of the uterine manipulator: patients who underwent MIS with a uterine manipulator had a 2.76-fold higher risk of recurrence compared to those treated by the open approach. Patients treated with MIS without the uterine manipulator had similar rates of relapse as those who underwent ORH [28]. The experts agreed that “the use of the uterine manipulator might be one of the main reasons for the poorer outcomes after minimally invasive surgery”. However, from the primary studies included in the three reviews, we were unable to determine the influence of the surgical technique.

Since the LACC trial had no unique preoperative technique for tumors <2 cm separately, the influence of tumor size on survival and recurrence rates remains a debated issue. Several groups have tried to evaluate the outcome for tumors <2 cm, but the data permit no unequivocal statement. In general, MIS appears to be as safe as ORH in this subgroup [28,29,30].

The interviewed gynecological experts emphasized the importance of sufficient lymph node dissection independent of the surgical technique. Zhao et al. reported significantly fewer pelvic lymph nodes retrieved with LRH compared with ORH, but Zhang et al. found no significant difference between RRH and ORH [21,22]. The limited body of data concerning a potential difference in prognosis between minimally invasive surgery and laparotomy based on the number of extracted lymph nodes also calls for further investigation.

According to the experts, pelvic lymph node dissection via MIS is effective when performed at highly qualified centers by skilled and well-trained surgeons. The most widely accepted explanation is that surgery by laparoscopy or robotics provides the surgeon with a better view of anatomical structures and surgical landmarks, which could be beneficial for the excision of lymphatic tissue. However, experts at the University of Kiel stated that “local recurrences within the lower pelvis, specifically in the area of the internal iliac artery” appear to be more frequent in minimally invasive surgery.

The existing published literature does not permit surgeons to adopt a specific procedure as the gold standard for the treatment of early-stage cervical cancer. However, none of the experts we interviewed were aware of a significantly higher recurrence rate with MIS compared to ORH.

The LACC trial, as an RCT, offers the highest level of evidence in the existing literature on the treatment of early-stage cervical cancer. The bias potential of non-randomized studies must be considered when comparing the existing data with the LACC trial.

The experts were unanimous as regards their obligation to inform patients about the results of the LACC trial [8]. In addition to the critical points of the LACC trial, patients should be informed about studies reporting contrary results, such as those of Chiva and Köhler et al., who mention an equivalent outcome for MIS without the use of a uterine manipulator and with the use of combined vaginal-assisted laparoscopic radical hysterectomy (VALRH) for protective vaginal closure [28,31]. The surgical approach should be selected on the basis of a shared decision-making process.

However, it should be noted that current studies reporting expert opinions on the issue are not concurrent with regard to the surgeon’s individual long-term experience. Not all questions raised by the LACC trial have been resolved. Well-designed and -structured studies will be needed in the near future in order to offer patients accurate advice for the treatment of early-stage cervical cancer [32].

## Figures and Tables

**Table 2:** Results of the LACC trial.

Outcome	MIS	ORH	MIS vs. ORH (95% CI)
Intention to treat (ITT)	*n* = 319	*n* = 312	
DFS at 4.5 years (%) (95% CI)	86.0 (79.7 to 90.4)	96.5 (92.7 to 98.4)	−10.6 (−16.4 to −4.7) *
Disease recurrence or death from cervical cancer; *n* (%)	27 (8.5)	7 (2.2)	HR 3.74 (1.63 to 8.58) **
Disease recurrence or death from any cause (PFS); *n* (%)	32 (10.0)	8 (2.6)	HR 3.88 (1.79 to 8.41)
Locoregional recurrence; *n* (%)	18 (5.6)	4 (1.3)	HR 4.26 (1.44 to 12.60)
Death from any cause; *n* (%)	19 (6.0)	3 (1.0)	HR 6.00 (1.77 to 20.30)
Death from cervical cancer; *n* (%)	14 (4.4)	2 (0.6)	HR 6.56 (1.48 to 29.00)
**Per-protocol (PP)**			
DFS at 4.5 years; (%) (95% CI), *n* = 289	87.1 (81.0 to 91.3)	97.6 (94.1 to 99.0)	−10.5 (−16.0 to −5.0)
Laparoscopic only; (%) (95% CI), *n* = 244	87.0 (80.5 to 91.5)	97.6 (94.1 to 99.0)	−10.6 (−16.4 to −4.7)
Robotic only; (%) (95% CI), *n* = 45	87.2 (64.0 to 95.9)	97.6 (94.1 to 99.0)	−10.4 (−24.7 to 3.9)

Overview of main endpoints and results of the LACC trial (Ramirez et al., 2018). A significantly poorer outcome was reported for MIS compared to ORH (e.g., DFS at 4.5 years 87.1% vs. 97.6%). Based on Table 1 of Ramirez 2018 [1]. * *p* = 0.87, test for non-inferiority; ** *p* = 0.002, test for superiority; CI = confidence interval; DFS = disease-free survival; HR = hazard ratio; ITT = intention to treat; LACC = laparoscopic approach to cervical cancer; MIS = minimally invasive surgery; ORH = open radical hysterectomy; PFS = progression-free survival; PP = per-protocol.

**Table 3:** LACC trial—Adverse events.

Outcome	MIS (*n* = 279)	ORH (*n* = 257)	MIS vs. ORH (95% CI)
Any AE; *n* (%)	164 (59)	136 (53)	5.9 (−2.5 to 14.3)
Intraoperative AE; *n* (%)	34 (12)	26 (10)	2.1 (−3.3 to 7.4)
Postoperative AE; *n* (%)	152 (54)	124 (48)	6.3 (−2.2 to 14.7)
Major AE; *n* (%)	50 (18)	41 (16)	2.0 (−4.4 to 8.3)
Serious AE; *n* (%)	39 (14)	30 (12)	2.3 (−3.3 to 8.0)
Duration of surgery (min); geometric mean (range)	216 (75 to 441)	187 (61 to 425)	*p* < 0.001 *
Estimated blood loss (mL); geometric mean (range)	101 (10 to 1500)	209 (10 to 2200)	*p* < 0.001 *
Length of hospital stay (days); median (range)	3 (0 to 72)	5 (1 to 69)	*p* = 0.002 *

Overview of adverse events reported in the LACC trial (Ramirez et al., 2018). ORH was associated with a poorer outcome in terms of the duration of surgery, estimated blood loss, and length of hospital stay. No significant difference was seen between MIS and ORH in regard to all other adverse events. Based on Tables 2 and 3 of Obermair et al., 2020 [23]. * No effect estimate reported. AE = adverse event; CI = confidence interval; LACC = laparoscopic approach to cervical cancer; min = minutes; MIS = minimally invasive surgery; mL = milliliter, ORH = open radical hysterectomy.

**Table 1:** Overview of included reviews.

	Greggi 2020 [20]	Zhang 2019 [22]	Zhao 2017 [21]
Search dates	NR *	February 2018	February 2016
Databases	NR *	Pubmed, Embase, Cochrane Library, and Web of Science English only	Medline, Web of Knowledge, Cochrane Library, and Chinese National Knowledge Infrastructure No language restrictions
Population	Cervical cancer (stage IA1 to stage IB)	Cervical cancer Studies in patients with malignant gynecological lesions other than cervical cancer were excluded.	Cervical cancer stage IA1 to IIA treated with radical hysterectomy with or without lymphadenectomy Radical trachelectomy, radical vaginal hysterectomy, abdominal assisted vaginal hysterectomy, and recurrent cervical cancer cases were excluded
Intervention	MIS	RRH	LRH
Comparators	ORH	LRH and ORH	ORH (abdominal)
Outcomes prespecified	NR *	Surgical outcomes (not specified)	Intra and postoperative outcomes (listed in outcomes reported)
Study designs	NR *	Prospective and retrospective cohort	Prospective and retrospective comparative
Quality assessment	NR	MINORS and Newcastle–Ottawa scale (only MINORS was reported and used to select studies for the meta-analysis, scores < 12 were excluded)	Newcastle–Ottawa scale
Number of studies	24 (MIS vs. ORH 5, LRH vs. ORH 14, RRH vs. ORH 3, LRH vs. RRH vs. ORH 2)	25, 13 included in MA (RRH vs. ORH 4, RRH vs. LRH 6, RRH vs. LRH or ORH 3)	23
Study design	MA (3), retrospective (17) and prospective (1) observational studies, epidemiological studies (3)	Cohort (9, with no details) Matched (4, with no details)	RCT (1), prospective (2), retrospective (matched case–control 2), retrospective (18)
Outcomes reported	DFS, OS, recurrence rate	DFS, OS, operating time, blood loss, transfusion, conversion, intraoperative complications, postoperative complications, length of hospital stay, retrieved lymph nodes, recurrence	Operating time, blood loss, transfusion rate, length of hospital stay, return to normal bowel activity, duration of bladder catheterization, intestinal injury, retrieved lymph nodes, length of follow-up, recurrence, specific types of complications and injury
Conclusion	ORH should be considered the standard of care. Patients should be counseled carefully before MIS, which can be considered safe only for sentinel lymph node mapping in a fertility-sparing setting and could be considered after preoperative conization and for small tumors, preventive surgical maneuvers, and in reference centers.	RRH was safe, effective, and comparable to ORH and LRH with respect to outcomes in regard of surgical trauma and postoperative recovery.	LRH was effective, safe, and superior to the abdominal approach in terms of the most essential short- and long-term surgical outcomes. For some terminal parameters, such as 5 year survival or mortality, more data will be needed.

Overview of included reviews and SR. References are compared by main characteristics. All references refer to early-stage cervical cancer. Greggi et al., a narrative review, included 24 primary sources; Zhao and Zhang, both SRs, included 25 and 23 primary sources, respectively. While Greggi et al. and Zhao compared minimally invasive surgery vs. open surgery, Zhang compared RRH vs. LRH and ORH. NR = not reported; MIS = minimally invasive surgery; RRH = robotic radical hysterectomy, LRH = laparoscopic radical hysterectomy; ORH = open radical hysterectomy; MA = meta-analysis; RCT = randomized controlled trial; DFS = disease-free survival; OS = overall survival; * = literature review without reporting of systematic literature review or pre-specified inclusion criteria.

**Table 4:** DFS, OS and recurrence outcomes for the LACC trial and the reviews.

	LACC [1,18,19]	Greggi 2020 [20]	Zhang 2019 [22]	Zhao 2017 [24]
DFS (HR/RR) (95% CI)	HR 3.74 (1.63 to 8.58), MIS vs. ORH	HR 0.97 (NR), *p* = 0.91. LRH vs. ORH	NR	NR
DFS up to 5 years (%)	86.0 vs. 96.5, difference −10.6 (−16.4 to −4.7), MIS vs. ORH	*Prospective studies:* 92.8 vs. 81.3, *p* = 0.03, MIS vs. ORH *Epidemiological studies:* 92.8 vs. 94.4, *p* = 0.499, LRH vs. ORH	*Matched studies:* 91.3 vs. 89.9, 90 vs. 89, 97 vs. 89, all RRH vs. LRH *Cohort studies:* 95.6 to 100 vs. 93.5 to 100 (depending on stage), 96.4 vs. 91.9, 89.7 vs. 89.8, all RRH vs. LRH; 89.7 vs. 84.6, RRH vs. ORH	NR
OS to 3 years (%) (HR/RR) (95% CI)	93.8 vs. 99.0; HR 6.00 (1.77 to 20.30), MIS vs. ORH	*Meta-analyses:* HR 0.98 (95% CI NR), *p* = 0.73, HR 0.91 (95% CI NR), *p* = 0.76, both LRH vs. ORH *Prospective studies:* 91.0 vs. 78.9, *p* = 0.026, MIS vs. ORH *Epidemiological studies:* 95.2 vs. 96.4, *p* = 0.451, LRH vs. ORH 90.9 vs. 94.7, *p* = 0.002, MIS vs. ORH	*Matched studies:* 100 vs. 83.4, RRH vs. LRH; 97 vs. 98, RRH vs. ORH *Cohort studies:* 96.6 vs. 95.9, RRH vs. LRH; 96.6 vs. 92.3, RRH vs. ORH	NR
Recurrence to 3 years (%) (HR/RR) (95% CI)	5.6 vs. 1.3, HR 4.26 (95% CI 1.44 to 12.60), MIS vs. ORH	*Meta-analyses:* 8.77 vs. 11.93 (95% CI NR) *p* = 0.2, 8.3% vs. 11.9 (95% CI NR), *p* = 0.16, both LRH vs. ORH *Prospective studies:* 15.1 vs. 14.4 (95% CI NR), *p* = 0.64, MIS vs. ORH *Epidemiological studies:* 6.1 vs. 5.7 (95% CI NR), *p* = NS, LRH vs. ORH	OR 0.85 (95% CI 0.58 to 1.27), 5 studies, RRH vs. ORH; OR 0.96 (95% CI 0.50 to 1.87), 7 studies, RRH vs. LRH	OR 0.74 (95% CI 0.49 to 1.13), 8 studies, LRH vs. ORH

Table 4 shows results of the references reported by Greggi et al., 2020, Zhang et al., 2019, Zhao et al., 2017, and Ramirez et al., 2018. Results are listed by the main endpoints of the LACC trial DFS, OS, and recurrence rates. Divergences were noted between the LACC trial and other sources: DFS to 5 years was 86.0 vs. 96.5% (MIS vs. ORH) in the LACC. Greggi et al.’s review of prospective studies showed 92.8 vs. 81.3% (MIS vs. ORH), epidemiological studies showed 92.8 vs. 94.4% (LRH vs. ORH). DFS = disease-free survival; HR = hazard ratio; RR = relative risk; CI = confidence interval; MIS = minimally invasive surgery; ORH = open radical hysterectomy; NR = not reported; LRH = laparoscopic radical hysterectomy; RRH = robotic radical hysterectomy; OS = overall survival.

**Table 5:** Intra and perioperative outcomes for the LACC trial and the reviews.

	LACC [1,23]	Greggi 2020 [20]	Zhang 2019 [22]	Zhao 2017 [21]
Estimated blood loss (mL) (geometric mean (range), MD (95% CI))	101 (10 to 1500) vs. 209 (10 to 2200), *p* < 0.001, MIS vs. ORH	NR	MD −322.59 (−502.75 to −142.43) 5 studies, RRH vs. ORH MD −22.25 (−81.38 to 36.87) 8 studies, RRH vs. LRH	MD −178.41 (−214.89 to −141.94), 13 studies, LRH vs. ORH
Duration of surgery (min) (geometric mean (range), MD (95% CI))	216 (75 to 441) vs. 187 (61 to 425), *p* < 0.001, MIS vs. ORH	NR	MD 36.07 (5.83 to 66.31) 6 studies, RRH vs. ORH MD 18.10 (−14.94 to 51.13) 9 studies, RRH vs. LRH	MD 43.68 (29.42 to 57.95), 15 studies, LRH vs. ORH
Blood transfusion (%) (OR (95% CI))	3.6 vs. 7.8, *p* = 0.03 *, MIS vs. ORH	NR	OR 0.19 (0.09 to 0.39) 6 studies, RRH vs. ORH OR 0.53 (0.16 to 1.75) 5 studies, RRH vs. LRH	OR 0.47 (0.30 to 0.73), 13 studies, LRH vs. ORH
Intraoperative complications (%) (OR (95% CI))	12.2 vs. 10.1, *p* = 0.45, MIS vs. ORH	NR	OR 0.52 (0.27 to 0.98) 5 studies, RRH vs. ORH OR 1.17 (0.44 to 3.10) 7 studies, RRH vs. LRH	OR 1.14 (0.68 to 1.93), 8 studies, LRH vs. ORH
Postoperative complications (%) (OR (95% CI))	59 vs. 53, *p* = 0.17, MIS vs. ORH	NR	OR 0.74 (0.45 to 1.22) 7 studies, RRH vs. ORH OR 0.66 (0.39 to 1.12) 9 studies, RRH vs. LRH	NR
Length of hospital stay (days) (median (range), MD (95% CI))	3 (0 to 72) vs. 5 (1 to 69), *p* = 0.002, MIS vs. ORH	NR	MD −2.71 (−3.74 to −1.68) 6 studies, RRH vs. open RRH MD −0.24 (−1.33 to 0.85) 9 studies, RRH vs. LRH	MD −3.17 (−4.06 to −2.29), 14 studies, LRH vs. ORH
**Retrieved lymph nodes (*n*)**	Median: 20 (15 to 26) vs. 21 (16 to 30), MIS vs. ORH	NR	MD −3.43 (−7.74 to 0.88) 6 studies, RRH vs. ORH MD 2.46 (−0.46 to 5.38) 9 studies, RRH vs. LRH	MD −1.65 (−3.19 to −0.11), 8 studies, LRH vs. ORH
**DVT/PE (%)** **(OR (95% CI))**	0.4 vs. 0, *p* = 0.64, MIS vs. ORH	NR	NR	OR 1.31 (0.48 to 3.57), 5 studies, LRH vs. ORH
**Ileus (%)** **(OR (95% CI))**	0 vs. 0.8, *p* = 0.35, MIS vs. ORH	NR	NR	OR 0.34 (0.12 to 0.91), 7 studies, LRH vs. ORH
**Lymphocele/lymphedema (%)** **(OR (95% CI))**	0 vs. 1.2, *p* = 0.2 (lymphocele), 0.4 vs. 0.8, *p* = 0.52 (lymphedema), MIS vs. ORH	NR	NR	OR 1.46 (0.49 to 4.36), 5 studies, LRH vs. ORH
**Duration of bladder catheterization (days) (MD (95% CI))**	Delay in bladder function (%), 4.7 vs. 5, *p* = 0.87, MIS vs. ORH	NR	NR	MD −1.69 (−2.83 to −0.55), 3 studies, LRH vs. ORH
**UTI (OR (95% CI))**	NR	NR	NR	OR 0.58 (0.22 to 1.57), 4 studies, LRH vs. ORH
**Ureteral/vaginal fistulas (OR (95% CI))**	Genitourinary fistula/stricture (%) 3.6 vs. 2.7, *p* = 0.57, MIS vs. ORH	NR	NR	OR 3.67 (1.08 to 12.47), 5 studies, LRH vs. ORH

Adverse events in the references reported by Greggi et al., 2020, Zhang et al., 2019, Zhao et al., 2017, and Ramirez et al., 2018. mL = milliliter; MD = mean deviation; CI = confidence interval; MIS = minimally invasive surgery; ORH = open radical hysterectomy; NR = not reported; RRH = robotic radical hysterectomy; LRH = laparoscopic radical hysterectomy; OR = Odds ratio; DVT/PE = deep vein thrombosis/ pulmonary embolism; UTI = urinary tract infection; * = intraoperative and/or postoperative transfusions.

## Supplementary Materials

The following are available online at https://www.mdpi.com/article/10.3390/jcm10173761/s1, Table S1: Full ROBIS assessments for each review.

## Appendix A

### Appendix A.1. Responses by Clinical Experts

#### Appendix A.1.1. Professor D. Bauerschlag, University Hospital Schleswig-Holstein, Campus Kiel, Germany

Based on your experience with the treatment of cervical cancer, could you see any differences between minimally invasive surgery vs. open radical hysterectomy with regard to risk of death?

- Based on my experience I do overview by fare more minimal invasive procedures than open radicals. However, I cannot report any differences in survival regarding the technique used.

Do you agree, that the results of the LACC trial, as a single multicenter randomized controlled trial, should overweigh the body of evidence from mostly retrospective observational studies?

- Although the LACC trail is a multicenter randomized trial it has some caveats lowering the power of its results. But since it is Level Ib evidence one should be cautious not considering these results when discussing the surgical procedure with the patient. To me the results of LACC overweigh the results from mostly retrospective observational studies.

Which consequences do the results of the LACC trial have on your daily practice?

- Patients need to be informed about the results of LACC during the informed consent procedure prior to treatment. Having said that, I would perform robotic assisted minimal invasive surgery in cases such as ≤FIGO Ia2, no evidence of lymphvascular invasion and no adenocarcinoma differentiation. Furthermore, we do have to inform the patient that radio-therapy is a valid option curing patients from low stage cervical cancer.

From your experience with the treatment of cervical cancer could you see any difference between minimally invasive surgery vs. open radical hysterectomy according to the recurrence rate?

- To me local recurrences within the lower pelvis, explicitly in the area of the internal iliac artery seems higher to me in minimal invasive surgery.

Do you agree that Cervical cancer cells stimulated by a CO_2_ pneumoperitoneum environment have an increased the ability to proliferate after a short period of inhibition?

- I doubt that CO_2_ plays a role in cell proliferation. However, I can imagine that the increased intra-abdominal pressure could potentially affect the tumor cell that way that residual cells are pushed into niches.

Do you evaluate these facts as critical weakness for the inclusion criteria at the LACC trial?

- The facts are weakening the LACC results but overall the results seem to be reliable.

#### Appendix A.1.2. Professor H. Ferreira, University Hospital Center of, Portugal

Do you agree, that the results of the LACC trial, as a single multicenter randomized controlled trial, should overweigh the body of evidence from mostly retrospective observational studies.

- The LACC trial outcomes should be taken into account critically and meticulously compared with other scientific validated studies that several oncological referral centres have already published. It may have a problematic premise and surgeon bias. The LACC trial seems to have methodology limitations, and the inclusion criteria are questionable (pe. surgeon proficiency criteria for minimally invasive surgery in the trial were only 10 cases and a total of 2 un-edited videos; the participation of countries where minimally invasive surgery has become the treatment of choice was low, and the LACC trial did not report the operational technique, etc.). The surgical related morbidity and mortality may be worse in the laparotomy arm. There is missing data in the LACC trial like surgical cohorts well balanced for all demographic variables, all tumours variables, stage, the overall number of lymph nodes removed, number of positive lymph nodes, post-treatment receipt of adjuvant radiation and chemotherapy, …

Which consequences do the results of the LACC trial have on your daily practice?

- After the LACC trial, we developed a new informed consent form in the department. The patients should be clearly informed and allowed to choose. If patients consent, we offer a minimally invasive surgical approach in cases of squamous cell carcinoma in stage IB1 cervical cancer with tumour size ≤ 2 cm. We’ve implemented routine vaginal closure before colpotomy (vaginal cuff from below, by laparoscopy using a linear stapler).

While new and reliable data comes in, the option has been more towards an open radical hysterectomy approach.

From your experience with the treatment of cervical cancer comparing minimally invasive surgery (laparoscopic radical hysterectomy and/or robotic radical hysterectomy) vs. open radical hysterectomy could you report any differences of intra-operative and post-operative complications?

- Our data and covering intra-operative complications do not report any significant difference between radical laparoscopic hysterectomy and open radical hysterectomy. Concerning postoperative complications, they have been more common in the open radical hysterectomy group (namely urinary retention, sexual and bowel dysfunction). Probably, because by laparoscopic/robotics approach, it is possible to identify better the anatomy, and a nerve-sparing approach is more feasible. Seeing better, we also can treat better. The pneumodissection offered by laparoscopy/robotics facilitates the access of the retroperitoneal space, where the lymphatic nodes are located.

Do you share the critique points, and do you believe that the pelvic lymph nodes dissection with minimal invasive surgery is technically more complicated?

- With a well-trained team and following the standard steps, MIS enhances the pelvic lymph node dissection. The surgeon has a better anatomical vision by laparoscopy or robotics and can be more efficient in the lymphatic tissue excision respecting anatomical and surgical landmarks. The precision offered by laparoscopy/robotics allows a more precise approach, sparing the nerves, the small vessels and even, in some cases, the lymphatics (eventually causing less lymphedema/lymphocysts).

#### Appendix A.1.3. Professor P. Hillemanns, University Hospital Hannover, Germany

Do you consider the LACC-trial as a valid study in spite of some weaknesses?

- The LACC study is a large international randomized study that included 631 patients in around 33 centres (USA, Central/South America, Eastern Europe, Australia, China, etc.).The question was whether laparoscopic radical hysterectomy is not inferior to open surgery. The study was terminated early because the data security committee showed a significant superiority of open surgery. The study is very informative regarding the question of which surgical procedure is better for early cervical cancer of up to 4 cm size. Due to the study design with randomization and multicentre approach in connection with the number of different design (retrospective, comparison with historical cohorts, one-arm studies, register and population avaluations).

In my opinion, the LACC study has the following points of criticism:

1. Based on the reported funding of $121.250 from Medtronic in the original publication, it must be doubted that sufficient funding for management, data collection and quality assurance with appropriate validation of the many parameters was possible.
2. The main point of criticism, in my opinion, is the failure to observational studies from the last two years, can have a significant influence on tumour cell dissemination and thus on the locoregional recurrence rate in laparoscopic radical hysterectomy. In particular, the use of the uterine manipulator and abdominal colpotomy are associated with an increased risk. The formation of a vaginal cuff over the portio is another protective maneuver.
3. The brief follow-up observation of the study can in principle be viewed as a point of criticism. It was shown that in the study evaluation in 2019, i.e., one year after the publication in the NEJM the HR decreased from 6.0 to 2.9. However, the differences are still clearly significant!
4. Some critics question the operative expertise of the respective centres, especially with reference to the learning curve of laparoscopic radical hysterectomy, which became established as a routine procedure worldwide only a few years before the start of the study.

In summary, I would like to refer to the statement by the AGE and AGE, published with me as first author in GebFra, and I still fully agree with the statement even 18 months after this publication.

Do you agree, that the result of the LACC trial, as a single multicentre randomized controlled trial, should overweigh the body of evidence from mostly retrospective observational studies?

- I am convinced that the validity of the only larger randomized multicentre study to date is very high and with regard to its evidence, outweighs the retrospective observational studies. The results of the retrospective observational studies are, however, generating hypotheses, so that, in my opinion, further larger multicentre randomized studies should urgently be initiated.

Which consequences do the results of the LASS trial have for your daily practice?

- In everyday clinical practice, our patients are very openly informed about the two methodological approaches in the treatment of early cervical cancer. We point out that the LACC study has the highest informative value and emphasize the significantly better results in terms of overall survival and disease-free survival. However, we also mention the operational and technical points of criticism of the study and refer to the publications by Professor Köhler and my senior physician, Professor Hertel, in the International Journal of Gynaecology Cancer 2019 and to the results of the SUCCOR study (Chiva, 2019). The retrospective evaluation of our laparoscopic radical hysterectomy compared to open surgery yielded similar high DFS/OS rates compared to the open LARRarm and the Köhler and Chiva-protectivemaneuver-arms.
  Nevertheless, we stress the power of an RCT in this question about the operative approach.

#### Appendix A.1.4. Professor N. Maass, University Hospital Schleswig-Holstein, Campus Kiel, Germany

Based on your experience with the treatment of cervical cancer, could you see any differences between minimally invasive surgery versus open radical hysterectomy?

- During the last ten years, we didn’t notice any difference between the two approaches in University Hospital Kiel, although we didn’t collect out data. In University Hospital Aachen, Germany in the early 2000s we noticed a difference. But at that time, we could not define the reason for the worse outcome after the minimally invasive surgery.

Do you agree, that the result of the LACC trial, as a single multicentre randomized controlled trial, should overweigh the body of evidence from mostly retrospective observational studies.

- The LACC trial by Ramirez et al. was a very well performed and well-structured study. The medical centres taking part had a high standard. We have to take the results seriously although smaller, mostly retrospective studies, inferior to the LACC trial, showed different results. In my opinion, every medical centre must keep an eye on their own outcome data.

Do you think, that the inconsistent use of the uterine manipulator and applied technic as well as the unequal experience of the surgeons of the partaking medical centres of the LACC trial had an influence of the results?

- I don’t think, that the different experience of the involved surgeons had an outstanding impact on the results of the LACC trial. The effect on the outcome was remarkable even with well trained and very experienced surgeons. The usage of the uterine manipulator might be one of the main reasons for the worse outcome data after minimally invasive surgery. One could easily imagine that tumour cells might migrate into the pelvis once the tumour is broken by the manipulator. Data from Köhler et al. support that concept.

Do you see CO_2_ pneumoperitoneum as a reason for the worse outcome after MIC?

- Trocar metastasis as well as CO_2_ must be considered as possible reasons for the worse outcome data after MIC.

Which consequences do the results of the LASS trial have for your daily practice?

- After the publication of the LACC trial one must inform the patient very well about the existing data and based on that, one should perform a shared decision making considering the advantages and disadvantages of both approaches. For patients with tumours > 2 cm we must offer the open radical hysterectomy, based on the findings of Ramirez et al. And one should avoid the uterine manipulator.

#### Appendix A.1.5. Dr. M. Elessawy, University Hospital Schleswig-Holstein, Campus Kiel, Germany

Do you believe that the surgical technique plays a leading role for the prognosis of the patients?

- The critical factor with the level of the experience of the surgeons remain underestimated in the literature, one of the drawbacks of the LACC trial is the absence of a unique parameter for the inclusion of the surgeons and difficulty to reach a unique evaluation of the quality of the surgery performed.
  Moreover, the different number of dissected pelvic lymph nodes with in the surgery shows the need for a universal definition, however the risks profile and the cofactors are different from one case to the other.
  The role presented by the Co2 demands a detailed investigation in the near future, however it increases the propensity for cervical cancer to implant.
  The uterine manipulator plays a role for increasing the recurrence rate at the vaginal vault, probably through invasion of the vaginal mucosa, resulting in the spread and spillage of the tumour cells.

Which consequences do the results of the LASS trial have for your daily practice?

- Since the publication of the results of the LACC study, we perform a detailed consultation of the patients showing the results published by the LACC study and the discrepancy to our individual results. The decision for the surgery is based on shared decision making with the help of decision panels which shows the different literature and experience. I emphasise the unknown role of the manipulator and the fact that also non vaginal vault pelvic recurrences occurred in the minimally invasive surgery group.
  The importance to have a standard uniform adequate staging examination of the patients before planning the entire treatment in order to avoid the discrepancy and the heterogenicity by risk of recurrence, which is one of the drawbacks of the LACC study, with variation and sometimes lack of presurgical assessment of the staging examination.
  The need for a more detailed decisions aid presented by the oncology society would results in a more confidence at the process of decision making and avoid any sort of prevalence by the centres, which emphasize the need to organize more training sessions to improve and retrain the training skills of the medical personals to perform those sophisticated consultation.

**Table A1:** Primary studies included in the reviews.

Studies	Greggi 2020 [20]	Zhang 2019 [22]	Zhao 2017 [21]
Abu-Rustum 2003 Gynecol Oncol			✓
Asciutto 2015 Acta Obstet Gynecol Scand		✓	
Boggess 2008 Am J Obstet Gynecol		✓	
Cao 2015 J Laparoendoc Adv Surg Tech A	✓		
Chen 2012 J Sanxi Med Univ			✓
Chen 2014 Int J Gynecol Cancer		✓	
Chiva 2019 ESGO conference	✓		
Choi 2012 Ann Surg Oncol			✓
Chong 2013 Int J Gynecol Cancer		✓	
Corrado 2016 Int J Gynecol Cancer		✓	
Corrado 2018 Int J Gynecol Cancer	✓		
Cusimano 2019 Am J Obstet Gynecol	✓		
Dao 2012 Matern Child Health Care China			✓
Desille 2013 Eur J Obstet Gynecol Reprod Biol		✓	
Diaz- Feijoo 2014 Gynecol Oncol		✓	
Ditto 2015 Eur J Surg Oncol	✓		✓
Diver 2017 J Minim Invasive Gynecol	✓	✓	
Doo 2019 Gynecol Oncol	✓		
Estape 2009 Gynecol Oncol		✓	✓
Frumovitz 2007 Obstet Gynecol			✓
Geisler 2010 Int J Gynecol CancerV		✓	
Ghezzi 2007 Gynecol Oncol			✓
Gil-Moreno 2019 J Minim Invasive Gynecol	✓		
Guo 2018 Onco Targets Ther	✓		
Kim 2019 Cancer Res Treat	✓		
Kim 2019a Gynecol Oncol	✓		
Kim 2019b Gynecol Oncol	✓		
Kim JY 2015 Anticancer Res		✓	
Kim TH 2015 Anticancer Res		✓	
Ko 2008 Gynecol Oncol		✓	
Kong 2014 Int J Gynecol Cancer			✓
Lambaudie 2010 Eur J Surg Oncol		✓	
Laterza 2016 Int J Gynecol Cancer			✓
Lee 2011 Eur J Obstet Gynecol Reprod Biol	✓		✓
Li 2013 Shaanxi Med J			✓
Lim 2019 Gynecol Minim Invasive Ther	✓		
Malzoni 2009 Ann Surg Oncol	✓		✓
Melamed 2018 N Eng J Med	✓		
Mendivil 2016 Surg Oncol	✓	✓	
Nam 2010 Int J Gynecol Cancer		✓	
Nam 2012 Ann Oncol	✓		
Nezhat 1992 Am J Obstet Gynecol		✓	
Paik 2019 Gynecol Oncol	✓		
Park 2013 J Surg Oncol			✓
Pellegrino 2017 Acta Biomed		✓	
Sert 2011 Gynecol Oncol		✓	✓
Sert 2016 Eur J Surg Oncol	✓	✓	
Shah 2017 J Gynecol Oncol	✓		
Simsek 2011 Eur J Gynaecol Oncol			✓
Soliman 2011 Gynecol Oncol		✓	✓
Tinelli 2011 Ann Surg Oncol		✓	
Toptas 2014 J Laparoendosc Adv Surg Tech A			✓
Uccella 2007 Gynecol Oncol			✓
Uppal 2019 J Clin Oncol	✓		
Vizzielli 2016 J Minim Invasive Gynecol		✓	
Wang 2015 BMC Cancer	✓		
Wright 2012 Gynecol Oncol			✓
Xiong 2013 Mod Med J China			✓
Yim 2014 Yonsei Med J		✓	
Zanagnolo 2016 Int J Gynecol Cancer		✓	
Zhao 2017 J Laparoendosc Adv Surg Tech A	✓		
Zhu 2013 Zhejiang J Trauma Surg			✓
Zhu 2015 Henan J Surg			✓
