# Supplementary material for: Challenges and Controversies in the Surgical Treatment of Cervical Cancer: Open Radical Hysterectomy versus Minimally Invasive Radical Hysterectomy
Source: J Clin Med. 2021 Aug 24;10(17):3761. doi: 10.3390/jcm10173761 (PMC8432133; doi:10.3390/jcm10173761)
Supplement: Supplementary file 1 [file jcm-10-03761-s001.zip › jcm-1301539-supplementary.pdf]

Supplementary Table S1: Full ROBIS assessments for each review

Greggi 2020			
**Domain 1: Study eligibility criteria**			
**Question**	**Evidence**	**Rating**	**Overall domain rating**
1.1 Did the review adhere to pre-defined objectives and eligibility criteria?		No information	Unclear risk<br><br>This is a literature review not a systematic review so the review questions and eligibility criteria were not stated
1.2 Were the eligibility criteria appropriate for the review question?		No information	
1.3 Were eligibility criteria unambiguous?		No information	
1.4 Were all restrictions in eligibility criteria based on study characteristics appropriate?		No information	
1.5 Were any restrictions in eligibility criteria based on sources of information appropriate?		No information	
**Domain 2: Identification and selection of studies**			
**Question**	**Evidence**	**Rating**	**Overall domain rating**
2.1 Did the search include an appropriate range of databases/ electronic sources for published and unpublished reports?		No information	Unclear risk
2.2 Were methods additional to database searching used to identify relevant reports?		No information	
2.3 Were the terms and structure of the search strategy likely to retrieve as many eligible studies as possible?		No information	
2.4 Were restrictions based on date, publication format, or language appropriate?		No information	
2.5 Were efforts made to minimise errors in selection of studies?		No information	

Greggi 2020			
**Domain 3: Data collection and study appraisal**			
**Question**	**Evidence**	**Rating**	**Overall domain rating**
3.1 Were efforts made to minimise error in data collection?		No information	Unclear risk
3.2 Were sufficient study characteristics available for both review authors and readers to be able to interpret the results?		No information	
3.3 Were all relevant study results collected for use in the synthesis?		No information	
3.4 Was risk of bias (or methodological quality) formally assessed using appropriate criteria?		No information	
3.5 Were efforts made to minimise error in risk of bias assessment?		No information	
**Domain 4: Synthesis and findings**			
**Question**	**Evidence**	**Rating**	**Overall domain rating**
4.1 Did the synthesis include all studies that it should?		No information	Unclear risk<br><br>As this was a literature review which did not follow predefined inclusion criteria it is not possible to judge whether all relevant studies were included in the synthesis
4.2 Were all predefined analyses followed or departures explained?		No information	
4.3 Was the synthesis appropriate given the nature and similarity in the research questions, study designs and outcomes across included studies?	A narrative synthesis was presented which was appropriate	Probably yes	
4.4 Was between-studies variation (heterogeneity) minimal or addressed in the synthesis?		No information	
4.5 Were the findings robust, e.g. as demonstrated through funnel plot or sensitivity analyses?		No information	
4.6 Were biases in primary studies minimal		No information	

Greggi 2020			
or addressed in the synthesis?			
**OVERALL RATING OF RISK OF BIAS**			
**Question**	**Evidence**	**Rating**	
Did the interpretation of findings address all of the concerns identified in domains 1 to 4?		No information	
Was the relevance of identified studies to the review's research question appropriately considered?		No information	
Did the reviewers avoid emphasizing results on the basis of their statistical significance?		No information	
**UNCLEAR RISK OF BIAS**<br>This was a literature review and so did not use systematic review methods so many methodological aspects were not relevant or unclear			

Purwanti 2019			
**Domain 1: Study eligibility criteria**			
**Question**	**Evidence**	**Rating**	**Overall domain rating**
1.1 Did the review adhere to pre-defined objectives and eligibility criteria?	The protocol was published on PRISMA	Probably Yes	Unclear risk<br><br>Abstract and poster only with limited information
1.2 Were the eligibility criteria appropriate for the review question?	Based on the protocol, the eligibility criteria seem appropriate	Probably Yes	
1.3 Were eligibility criteria unambiguous?	Full details of eligible outcomes were not reported	No information	
1.4 Were all restrictions in eligibility criteria based on study characteristics appropriate?	No details	No information	
1.5 Were any restrictions in eligibility criteria based on sources of information appropriate?	The exclusion criteria suggest possible language restrictions (languages which the reviewers could not translate) but there were no further details	No information	
**Domain 2: Identification and selection of studies**			
**Question**	**Evidence**	**Rating**	**Overall domain rating**
2.1 Did the search include an appropriate range of databases/ electronic sources	Pubmed and SCOPUS were searched but no further details were provided.	Probably No	High risk

Purwanti 2019			
for published and unpublished reports?			Two databases were searched, and selection was performed by one reviewer.
2.2 Were methods additional to database searching used to identify relevant reports?	No details	No information	
2.3 Were the terms and structure of the search strategy likely to retrieve as many eligible studies as possible?	No details	No information	
2.4 Were restrictions based on date, publication format, or language appropriate?	Searches were not restricted by language.	Probably Yes	
2.5 Were efforts made to minimise errors in selection of studies?	Study selection was performed by one reviewer.	No	
**Domain 3: Data collection and study appraisal**			
**Question**	**Evidence**	**Rating**	**Overall domain rating**
3.1 Were efforts made to minimise error in data collection?	Data extraction was conducted independently by three reviewers	Yes	Unclear risk<br><br>There was no information about the included studies
3.2 Were sufficient study characteristics available for both review authors and readers to be able to interpret the results?	No details	No information	
3.3 Were all relevant study results collected for use in the synthesis?	No details	No information	
3.4 Was risk of bias (or methodological quality) formally assessed using appropriate criteria?	Risk of bias was assessed with the Newcastle Ottawa tool.	Probably Yes	
3.5 Were efforts made to minimise error in risk of bias assessment?	No details	No information	
**Domain 4: Synthesis and findings**			
**Question**	**Evidence**	**Rating**	**Overall domain rating**
4.1 Did the synthesis include all studies that it should?	No details	No information	Unclear risk<br><br>There were no details about the included or results of pairwise meta-analyses and
4.2 Were all predefined analyses followed or departures explained?	No details	No information	
4.3 Was the synthesis appropriate given the nature and similarity in the research questions, study designs and	Possibly not given that it contained observational studies but there were no further details	No information	

Purwanti 2019			
outcomes across included studies?			heterogeneity assessments
4.4 Was between-studies variation (heterogeneity) minimal or addressed in the synthesis?	The protocol mentions assessment of heterogeneity and subgroup analyses, but no results were reported	No information	
4.5 Were the findings robust, e.g. as demonstrated through funnel plot or sensitivity analyses?	The protocol mentions publication bias tests, but no results were reported	No information	
4.6 Were biases in primary studies minimal or addressed in the synthesis?	No details	No information	
**OVERALL RATING OF RISK OF BIAS**			
**Question**	**Evidence**	**Rating**	
Did the interpretation of findings address all of the concerns identified in domains 1 to 4?	No discussion as only an abstract/poster but the authors did acknowledge the limitations of including observational studies.	No	
Was the relevance of identified studies to the review's research question appropriately considered?		No	
Did the reviewers avoid emphasizing results on the basis of their statistical significance?		Yes	
**UNCLEAR RISK OF BIAS**<br>This review was reported as an abstract and poster with limited information			

Zhang 2019			
**Domain 1: Study eligibility criteria**			
**Question**	**Evidence**	**Rating**	**Overall domain rating**
1.1 Did the review adhere to pre-defined objectives and eligibility criteria?	No protocol was reported but the eligibility criteria were clear	Probably Yes	High risk<br><br>Outcomes were not clearly defined, and studies were selected for further analysis based on their quality score
1.2 Were the eligibility criteria appropriate for the review question?	The eligibility criteria were appropriate	Probably Yes	
1.3 Were eligibility criteria unambiguous?	Outcomes were not defined only described as “interested surgical outcomes”	No	
1.4 Were all restrictions in eligibility criteria based on study characteristics appropriate?	Inclusion was also based on study quality (< 12 points on the MINORS scale were excluded)	Probably No	

Zhang 2019			
1.5 Were any restrictions in eligibility criteria based on sources of information appropriate?	All restrictions were relevant for the review question	Probably Yes	
**Domain 2: Identification and selection of studies**			
**Question**	**Evidence**	**Rating**	**Overall domain rating**
2.1 Did the search include an appropriate range of databases/ electronic sources for published and unpublished reports?	PubMed, Embase, the Cochrane Library, and Web of Science were searched.	Probably Yes	High risk<br><br>The full search strategy was not reported and only studies published in English were included.
2.2 Were methods additional to database searching used to identify relevant reports?	References of the included studies were searched.	Yes	
2.3 Were the terms and structure of the search strategy likely to retrieve as many eligible studies as possible?	Only keywords were reported, not the full search strategy	No information	
2.4 Were restrictions based on date, publication format, or language appropriate?	Only studies published in English were included	Probably No	
2.5 Were efforts made to minimise errors in selection of studies?	Two reviewers independently performed study selection	Yes	
**Domain 3: Data collection and study appraisal**			
**Question**	**Evidence**	**Rating**	**Overall domain rating**
3.1 Were efforts made to minimise error in data collection?	Two reviewers performed the data collection.	Probably Yes	High risk<br><br>There was a lack of detail about the study participants and there were no study characteristics or results reported for the 13 studies excluded from the meta-analysis.
3.2 Were sufficient study characteristics available for both review authors and readers to be able to interpret the results?	Details of patient age and surgical stage were not reported. No data were reported for the 13 studies not included in the meta-analysis.	No	
3.3 Were all relevant study results collected for use in the synthesis?	All relevant study results appear to have been included	Probably Yes	
3.4 Was risk of bias (or methodological quality) formally assessed using appropriate criteria?	Risk of bias was assessed using two different tools, MINORS for inclusion screening and the Newcastle-Ottawa tool (although these results were not reported)	Probably Yes	
3.5 Were efforts made to minimise error in risk of bias assessment?	Two investigators conducted the risk of bias assessment.	Probably Yes	

<table>
<tr><th colspan="4">Zhang 2019</th></tr>
<tr><th colspan="4">Domain 4: Synthesis and findings</th></tr>
<tr><th>Question</th><th>Evidence</th><th>Rating</th><th>Overall domain rating</th></tr>
<tr><td>4.1 Did the synthesis include all studies that it should?</td><td>Intra- and postoperative outcomes were pooled in a meta-analysis, but overall and disease-free survival were presented in a narrative synthesis which was appropriate as the outcomes were reported in different ways</td><td>Probably Yes</td><td rowspan="6">High risk

Different study designs were pooled, statistical heterogeneity was high and not explored through further analyses.</td></tr>
<tr><td>4.2 Were all predefined analyses followed or departures explained?</td><td>The analyses followed those specified in the methods</td><td>Probably Yes</td></tr>
<tr><td>4.3 Was the synthesis appropriate given the nature and similarity in the research questions, study designs and outcomes across included studies?</td><td>Different study designs were pooled (prospective and retrospective) which is not appropriate</td><td>Probably No</td></tr>
<tr><td>4.4 Was between-studies variation (heterogeneity) minimal or addressed in the synthesis?</td><td>Statistical heterogeneity was high for many analyses which was not explored.</td><td>No</td></tr>
<tr><td>4.5 Were the findings robust, e.g. as demonstrated through funnel plot or sensitivity analyses?</td><td>No sensitivity analyses or funnel plots were provided</td><td>No</td></tr>
<tr><td>4.6 Were biases in primary studies minimal or addressed in the synthesis?</td><td>Biases were not addressed in the synthesis.</td><td>No</td></tr>
<tr><th colspan="4">OVERALL RATING OF RISK OF BIAS</th></tr>
<tr><th>Question</th><th>Evidence</th><th colspan="2">Rating</th></tr>
<tr><td>Did the interpretation of findings address all of the concerns identified in domains 1 to 4?</td><td>The authors acknowledged heterogeneity but no other limitations of the review.</td><td colspan="2">No</td></tr>
<tr><td>Was the relevance of identified studies to the review's research question appropriately considered?</td><td></td><td colspan="2">Probably No</td></tr>
<tr><td>Did the reviewers avoid emphasizing results on the basis of their statistical significance?</td><td>Only half the studies were included in the meta-analysis, no details and results were reported for the other studies</td><td colspan="2">No</td></tr>
<tr><td colspan="4">HIGH RISK OF BIAS
Methodological issues appear evident in the conduct of this review for all domains. Critically authors disregard the different stages of cervical carcinoma and the role that this variable may play in the clinical outcomes.</td></tr>
</table>

Zhao 2017			
**Domain 1: Study eligibility criteria**			
**Question**	**Evidence**	**Rating**	**Overall domain rating**
1.1 Did the review adhere to pre-defined objectives and eligibility criteria?	No protocol was reported but the eligibility criteria were reported	Probably Yes	Low risk<br><br>The definition of the eligibility criteria appears appropriate and there were no restrictions
1.2 Were the eligibility criteria appropriate for the review question?	Population, interventions, outcomes, and study designs were specified	Probably Yes	
1.3 Were eligibility criteria unambiguous?	Eligibility criteria were clear	Probably Yes	
1.4 Were all restrictions in eligibility criteria based on study characteristics appropriate?	Publications not reporting specified outcomes were excluded	Yes	
1.5 Were any restrictions in eligibility criteria based on sources of information appropriate?	No restrictions are reported.	Yes	
**Domain 2: Identification and selection of studies**			
**Question**	**Evidence**	**Rating**	**Overall domain rating**
2.1 Did the search include an appropriate range of databases/ electronic sources for published and unpublished reports?	Medline, Web of Knowledge (WOK), Cochrane Library and Chinese National Knowledge Infrastructure (CNKI).	Probably Yes	High risk<br><br>The search strategy does not appear sufficiently sensitive.
2.2 Were methods additional to database searching used to identify relevant reports?	References of the included studies were searched.	Yes	
2.3 Were the terms and structure of the search strategy likely to retrieve as many eligible studies as possible?	The search strategy was limited and did not allow for truncation.	Probably No	
2.4 Were restrictions based on date, publication format, or language appropriate?	No language restrictions were reported	Probably Yes	
2.5 Were efforts made to minimise errors in selection of studies?	Two reviewers independently performed study selection.	Yes	
**Domain 3: Data collection and study appraisal**			
**Question**	**Evidence**	**Rating**	**Overall domain rating**
3.1 Were efforts made to minimise error in data collection?	Data extraction was performed independently by two reviewers	Yes	High risk

Zhao 2017			
3.2 Were sufficient study characteristics available for both review authors and readers to be able to interpret the results?	There was a lack of information about the included studies, particularly age and cancer stage.	Probably No	There was a lack of information about the study characteristics.
3.3 Were all relevant study results collected for use in the synthesis?	Relevant results appear to have been collected	Probably Yes	
3.4 Was risk of bias (or methodological quality) formally assessed using appropriate criteria?	Risk of bias was assessed with the Newcastle Ottawa tool.	Probably Yes	
3.5 Were efforts made to minimise error in risk of bias assessment?	Two investigators conducted the risk of bias assessment.	Yes	
**Domain 4: Synthesis and findings**			
**Question**	**Evidence**	**Rating**	**Overall domain rating**
4.1 Did the synthesis include all studies that it should?	All relevant studies appear to have been included in the synthesis	Probably Yes	High risk<br><br>Different study designs were pooled, statistical heterogeneity was high and not explored through further analyses.
4.2 Were all predefined analyses followed or departures explained?	Authors appear to adhere to the planned analysis methods	Yes	
4.3 Was the synthesis appropriate given the nature and similarity in the research questions, study designs and outcomes across included studies?	Different study designs were pooled (prospective and retrospective) which is not appropriate	Probably No	
4.4 Was between-studies variation (heterogeneity) minimal or addressed in the synthesis?	Statistical heterogeneity was high for some outcomes and this was not explored.	No	
4.5 Were the findings robust, e.g. as demonstrated through funnel plot or sensitivity analyses?	No sensitivity analyses or funnel plots were provided	No	
4.6 Were biases in primary studies minimal or addressed in the synthesis?	Biases were not addressed in the synthesis.	No	
**OVERALL RATING OF RISK OF BIAS**			
**Question**	**Evidence**	**Rating**	
Did the interpretation of findings address all of the concerns identified in domains 1 to 4?	The authors acknowledged possible publication bias, limitations with study designs and heterogeneity.	No	
Was the relevance of identified studies to the		Probably yes	

Zhao 2017		
review's research question appropriately considered?		
Did the reviewers avoid emphasizing results on the basis of their statistical significance?		Probably yes
**HIGH RISK OF BIAS**<br>The search strategy was limited so some studies may have been missed. There was a lack of detail about the included studies and the results of the meta-analysis may not be reliable due to the pooling of prospective and retrospective studies and high levels of statistical heterogeneity.		
